# Are We Missing the Environmental Factors in AI-Based Fall Risk Models?: A Systematic Review

**DOI:** 10.21203/rs.3.rs-8723907/v1

**Published:** 2026-02-02

**Authors:** Jiyoun Song, Boeun Kim, Min-Jeoung Kang, Shuxuan Li, Lingjie Liu, Wonkyung Jung

**Affiliations:** University of Pennsylvania School of Nursing; University of Iowa College of Nursing; Harvard Medical School; University of Pennsylvania School of Social Policy and Practice; University of Pennsylvania School of Engineering; Boston College Connell School of Nursing

**Keywords:** Fall Risk Assessment, Predictive Modeling, Environmental Factors, Systematic Review, Nursing Informatics

## Abstract

**Background:**

Falls commonly occur in home environments where environmental conditions can contribute to fall risk. Identification and mitigation of environmental hazards are critical components of fall prevention. However, artificial intelligence (AI)-based fall prediction models have largely focused on individual-level predictors, with limited attention to home environmental hazards despite their modifiable role in fall risk.

**Objective:**

To systematically review how environmental factors are incorporated into existing AI-based fall risk prediction models and summarize reported AI approaches and model performance among community-dwelling older adults.

**Methods:**

This systematic review followed PRISMA guidelines. Six electronic databases (PubMed, Embase, CINAHL, Cochrane Library, Web of Science, and Scopus) were searched from inception through December 2025. Eligible studies applied AI-based models to predict falls among older adults in community settings and incorporated environmental factors as model inputs.

**Results:**

Of more than 10,000 records identified, nine studies met final inclusion criteria. Six used supervised machine learning with structured data, while three employed computer vision or robotics-based approaches. Environmental factors were heterogeneously represented, ranging from checklist-based indicators to sensor- and vision-derived measures. When included, environmental features contributed meaningful information by improving discrimination or identifying actionable home hazards (AUC-ROC ranged from 0.67 to 0.76).

**Conclusion:**

Environmental factors remain underemphasized in AI-based fall prediction models. Greater integration of standardized and context-aware environmental information may enhance the relevance and preventive utility of AI-based fall risk prediction in community settings.

## Introduction

Falls pose a critical challenge to healthy aging and remain one of the leading causes of injury among community-dwelling older adults.^1^ The incidence of falls continues to rise as the global population ages, with approximately one in four older adults experiencing at least one fall each year.^1^ Among older adults, falls are associated with serious physical, psychological, and social consequences, including fractures, traumatic brain injuries, functional decline, and long-term disability.^2,3^ Older adults aged 75 years and older experience the highest rates of fall-related hospitalization and are particularly vulnerable to prolonged recovery and subsequent loss of independence.^4^ These outcomes directly undermine well-being and healthy aging and contribute to greater healthcare utilization, long-term care needs, and economic burden.^5^

Recent advances in artificial intelligence (AI) and machine learning (ML) have generated growing interest in their application to fall risk prediction and prevention. A rapidly expanding body of literature has explored AI-driven approaches for fall prediction, including machine learning algorithms, deep learning models, and sensor- or wearable-based systems.^6–9^ Traditionally, fall risk assessment has relied on standardized clinical tools and screening instruments developed to identify individuals at elevated risk. Commonly used tools include the Berg Balance Scale (BBS) to assess functional balance,^10^ Functional Reach Test, and various fall risk questionnaires that assess fall history, mobility limitations, medication use, and comorbidities.^11^ These instruments have been widely implemented in clinical and community settings given their simplicity and straightforward administration. Data derived from these traditional assessment tools have also been used as input features in AI–based fall prediction models, either independently or in combination with additional clinical, functional, and sensor-derived variables.^12^ However, despite their widespread adoption, traditional fall risk assessment tools demonstrate significant limitations in capturing dynamic and environmental determinants of fall risk

Falls result from the interaction of multiple factors, including individual characteristics, physical and psychological impairments, and environmental conditions.^4,13^ Accurate fall prediction, therefore, requires approaches capable of integrating comprehensive, diverse and interrelated risk factors. While individual-level risk factors (e.g., age, comorbidities, medication use, and prior falls) and functional-level risk factors (e.g., balance, mobility, and functional limitations) have been extensively studied,^14,15^ home environmental hazards remain relatively understudied despite being an integral and modifiable component of fall risk among community-dwelling older adults. Notably, a majority of falls occur at home, contributing to 79.2% of fall-related emergency department visits,^16,17^ underscoring the central role of home settings in fall prevention efforts. Common hazards include inadequate lighting, slippery surfaces, loose rugs, unsafe handrails, and cluttered pathways,^4,18^ which often interact with age-related functional limitations, increasing fall risk and highlighting the need for systematic approaches to environmental risk identification and mitigation.

AI-based models offer unique advantages in handling complex, high-dimensional data and identifying non-linear interactions among multiple risk factors. Despite these advances, it remains unclear to what existing AI-based fall prediction models account for environmental risk factors which play a modifiable role in fall risk. To address this knowledge gap, this study aims to systematically synthesize the existing literature on the inclusion and representation of environmental factors in AI-based fall risk prediction models, and summarize the algorithms and performance metrics reported in studies of falls among community-dwelling older adults.

## Methods

This systematic review followed the guidelines of Preferred Reporting Items for Systematic Reviews and Meta-Analyses (PRISMA)^19^ The protocol was registered in the PROSPERO (CRD420261279513).

### Eligibility Criteria

#### (1) Inclusion Criteria

Studies were eligible for inclusion if they met the following criteria: (1) focused on community-dwelling older adults aged 60 and above from any country; (2) utilized AI or technology-based models to assess, predict, or prevent fall risks, including machine learning models or mobile health technologies; (3) assessed or predicted fall risk as a primary outcome; (4) incorporated environmental factors as one of the input variables or predictors in the model; (5) were data-based primary studies, including model development, validation, and evaluation studies reporting empirical data on model outcomes, accuracy, or effectiveness; and (6) were written in English.

#### (2) Exclusion Criteria

Studies were excluded if they: (1) focused on institutionalized populations such as nursing home residents; (2) employed non-technology or non-AI-based approaches, such as manual assessments or traditional fall risk screening methods; (3) were qualitative studies, dissertations, conference abstracts, editorials, commentaries, or literature reviews.

### Search Strategy

The literature search strategy was developed in collaboration with a librarian and conducted across six electronic databases (i.e., PubMed, Embase, CINAHL, Cochrane Library, Web of Science, and Scopus) on December 19, 2025. In addition, a manual review of the reference lists of included studies was conducted to identify additional eligible articles. No publication date limits were applied to ensure comprehensive retrieval of all relevant articles.

A comprehensive search methodology was designed using both free-text keywords and Medical Subject Headings (MeSH) terms (**Appendix 1**). The search strategy incorporated three major concept groups combined with Boolean operators: (1) Falls-related terms (e.g., “Accidental Falls”[MeSH], fall, falls, falling, fallen); (2) AI and technology-related terms (e.g., “Artificial Intelligence”[Mesh] machine learning, deep learning, telemedicine, digital health, mHealth, sensors, smart home technology); and (3) Older adult-related terms (e.g., aged[MeSH], older adult, elder*, senior*, geriatric*).

### Study Selection and Data Extraction

Search results were imported into Covidence (Veritas Health Innovation, Melbourne, Australia), and duplicates were removed prior to screening. Four reviewers [JS, BK, MK, and WJ] conducted a two-stage screening process. In the title and abstract screening stage, two reviewers independently assessed each record, and potentially relevant articles advanced to full-text review. During the full-text assessment, two reviewers again independently evaluated each article against eligibility criteria. At both stages, disagreements were resolved through consensus with a third reviewer who had not participated in the initial assessment.

In order to finalize the form, two authors created a standardized data extraction form and pilot-tested it on one article before finalizing the form. Each included study was independently extracted by two reviewers, with discrepancies resolved by a third reviewer. Extracted information included publication details (author, year, country, data source), study characteristics (aim, design, sample size, inclusion/exclusion criteria, recruitment), details of the AI model (type of approach, algorithms used, input variables), fall risk factors (including environmental factors and how they were measured) and model performance metrics (accuracy, sensitivity, specificity, AUC-ROC, F-score). Study limitations and potential conflicts of interest were also recorded.

Findings were synthesized into evidence tables summarizing study characteristics, AI approaches and risk factors, and model performance outcomes.

### Quality Appraisal

The methodological quality of included observational studies was assessed using the National Heart, Lung, and Blood Institute (NHLBI) Quality Assessment Tool for Observational Cohort and Cross-sectional Studies.^20^ The tool consists of 14 criteria evaluating clarity of research objectives, definition of the study population, participation rates, consistency of recruitment methods, adequacy of sample size justification, temporal relationship between exposure and outcome measurement, validity and reliability of exposure and outcome measures, blinding of outcome assessors, completeness of follow-up, and control for confounding variables. Each study was independently appraised by two reviewers, with discrepancies resolved through discussion with a third reviewer.

### Ethical Considerations

This systematic review utilized only publicly available published data and did not involve human subjects. Therefore, institutional review board (IRB) approval was not required.

## Results

**Figure 1:**
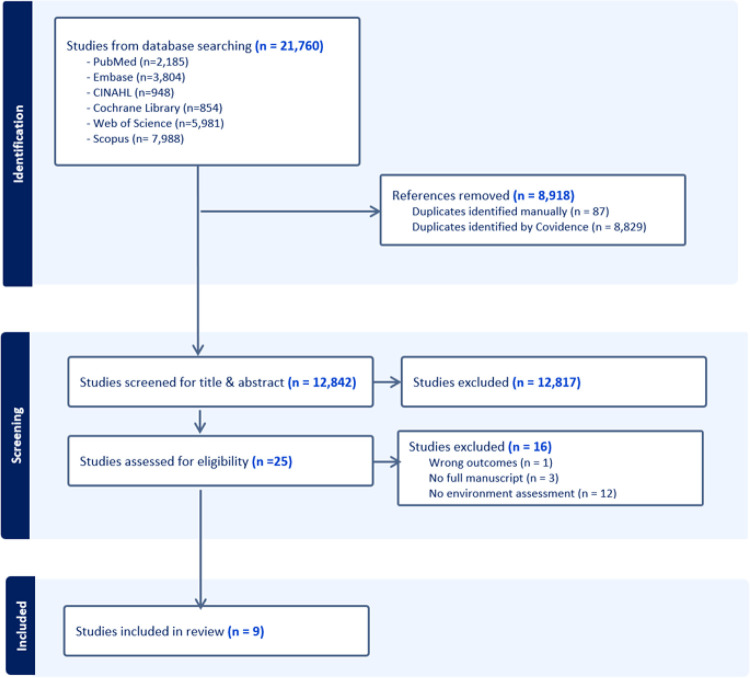
An Overview of the Review Process (PRISMA Diagram)

An overview of the review process is depicted in Fig. 1. A total of 21,760 articles were identified through the initial literature search, including PubMed (n = 2,185), Embase (n = 3,804), CINAHL (n = 948), Cochrane Library (n = 854), Web of Science (n = 5,981), and Scopus (n = 7,988). No additional studies were identified through manual searching of reference lists and research reports. After the removal of duplicate articles (n = 8,918), the remaining articles (n = 12,842) were screened. Of these, 12,817 were excluded after title and abstract review. During the full-text screening, an additional 16 articles were excluded for not meeting the inclusion criteria. Throughout the exclusion process, the majority of excluded studies did not include environmental risk factors, which were a key inclusion criterion. Consequently, a total of nine studies were included.

**Figure 2:**
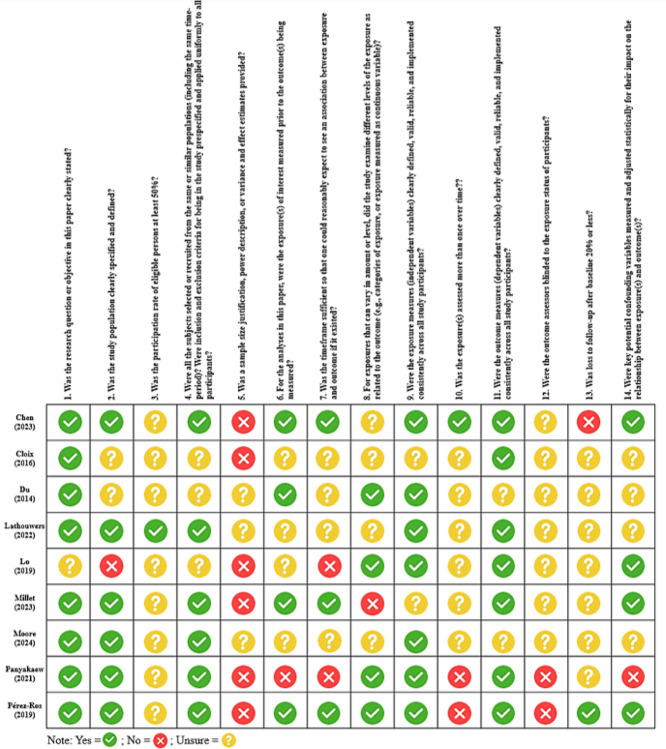
Quality Appraisal

Overall, the methodological quality of included studies was variable (Fig. 2). Most studies clearly stated research objectives and adequately defined study populations, but common limitations included incomplete reporting of sample size justification, limited handling of confounding, and insufficient information on exposure and outcome measurement reliability. Studies using vision- and sensing-based approaches, which rely on unstructured visual or depth data captured in real-world environments, showed greater uncertainty across several quality domains compared with structured-data–based machine learning studies, which used predefined, tabular inputs.

### Overview of Included Studies

Table 1 summarized the overview of included studies. The included studies were conducted across multiple countries, with the majority originating from the United States^21,22^ and European countries (Belgium,^23^ Switzerland,^24^ Spain,^25^ and the United Kingdom^26^), alongside representation from Asian countries including China^27^ and Thailand.^28^

Six studies (66.7%) used structured datasets, such as electronic health record (EHR), home health assessments or community-based registries, to train machine-learning models to predict future falls.^21,23,25,27–29^ In these studies, longitudinal follow-up periods and large samples were used to evaluate predictive performance across a wide range of demographic, clinical, and environmental variables. These included studies encompassed diverse populations with sample sizes ranging from 304 to 59,028 participants. The largest study by Lo et al.^21^ analyzed data from 59,028 unique patients aged over 65 years using retrospective EHR with available OASIS-C assessment and MAHC-10 fall risk assessment data. Lathouwers et al.^23^ examined 82,580 community-dwelling older adults aged 60 and over in Belgium, recruited through stratified random sampling by sex and age from census data; after excluding missing data, 24 input features and 33,346 entries remained for analysis.

In contrast, three studies used sensor- or computer-vision–based experimental designs.^22,24,26^ Du et al. employed robotics-enabled environmental screening incorporating luminosity and spatial measurements,^22^ while Cloix et al. validated depth-based stair detection using controlled motion capture settings.^24^ Moore et al. developed AI algorithms within simulated environments or structured testing conditions to detect environmental hazards or risky activities related to falls.^26^ These three studies did not provide detailed participant recruitment or demographic information.

### AI-based Fall Risk Prediction Models

Table 2 summarizes the characteristics of AI-based fall risk prediction models included in this review, including the type of AI algorithms used, environmental and non-environmental risk factors incorporated, reported model performance metrics, and key study limitations.

Six studies (66.7%) applied supervised machine learning models to structured clinical, functional, or survey data. Commonly used algorithms included logistic regression, random forest, and gradient boosting–based ensemble methods such as XGBoost, AdaBoost, and LightGBM. Several studies evaluated multiple classifiers with ensemble approaches. One study additionally incorporated natural language processing to extract features from narrative clinical text before model training.

Three studies focused on vision- or robotics-based approaches to environmental perception rather than direct fall outcome prediction. These studies employed techniques such as simultaneous localization and mapping (SLAM), depth-based detection pipelines, and deep learning object detection models (e.g., YOLOv8) to identify environmental features and hazards in home-like settings.

#### (1) Incorporation of Environmental Risk Factors

The included studies identified a wide range of environmental hazards at home that contribute to fall risk, though methods and comprehensiveness of environmental assessments varied considerably.

##### a. Structural and Housing Characteristics

Lathouwers et al.^23^ identified housing issues, housing change (i.e., homeownership or home type), and environmental vulnerability as significant predictors for fall occurrence, though specific measurement methods were not reported. Chen et al.^27^ incorporated multiple housing characteristics into both fall prediction and fall-related injury prediction models through structured inquiry, including building structure (reinforced concrete versus other materials), presence of handicapped facilities, kitchen and flush toilet availability, Internet access, house tidiness (clear versus unclear), and house temperature (hot, neutral, cold). Millet et al.^29^ assessed home-related features, including the presence of stairs, home accessibility, and elevator availability.

##### b. Specific Environmental Risk Factors

Perez-Ros et al.^25^ documented specific hazards through nurse-administered geriatric assessment, including lack of stair handrails, poor stair design, lack of bathroom grab bars, dim lighting or glare, obstacles and tripping hazards, slippery or uneven surfaces, and improper use of assistive devices. Lo et al.^21^ assessed environmental safety issues via structured assessment items completed by home health care clinicians, including clutter or obstacles, inadequate lighting, unsafe flooring, and lack of assistive devices or improper use. Du et al.^22^ focused on lighting-related hazards, including poor lighting, cluttered or narrow spaces, obstructed walkways, and limited visibility, and established quantitative luminosity thresholds that were displayed via a color-coded interface. Moore et al.^26^ identified 14 distinct environmental hazard categories using computer vision: stairs, doorways, showers, sinks, toilets, tables, beds, signage, chairs, animals, wet surfaces, mats/rugs/carpets, generic obstacles, and raised curbs. Panyakaew et al.^28^ integrated environmental components into validated mobility and balance scales, including sweeping the floor, reaching on tiptoes, walking in a crowded mall, and walking up/down stairs and ramps, using activities that directly reflect environmental challenges.

#### (2) Integration with Non-Environmental Risk Factors

Across studies, commonly integrated domains included demographic characteristics, clinical conditions, functional status, cognitive and psychological factors, medication use, and social or socioeconomic variables.^21,23,25,27^ Several models combined fall risk factors including age, prior falls, gait and balance impairments, chronic diseases, sensory deficits, and polypharmacy with psychosocial variables (e.g., depressive symptoms, fear of falling, social vulnerability) and functional dependence measures (Activities of Daily Living [ADL]/Instrumental Activities of Daily Living [IADL]).^21,29^ Some studies further incorporated disease-specific factors (e.g., Parkinson’s disease severity and motor complications) or physiological and performance-based measures (e.g., gait speed, grip strength, laboratory values).^26,28^

### AI Model Performance

Among studies using supervised machine learning on structured data, discrimination was generally modest to good. Reported AUC-ROC values ranged from approximately 0.67 to 0.76, with Chen et al.^27^ achieving the highest performance using logistic regression (AUC-ROC 0.739 for falls and 0.757 for fall-related injuries). Random forest–based models frequently outperformed traditional scoring tools, as demonstrated by Lo et al.^21^ (0.62 vs. lower baseline performance), although precision remained limited due to highly imbalanced outcomes. Perez-Ros et al.^25^ reported specificity exceeding 98% but sensitivity below 20%, indicating strong ability to rule in high-risk individuals but limited capacity to identify all fallers. Ensemble and gradient boosting approaches (e.g., XGBoost, LightGBM, bagging random forest) generally improved robustness and balance across performance metrics, with Millet et al.^29^ reporting the best overall performance for recurrent fall prediction (AUC ≈ 0.75; accuracy ≈ 76%).

Studies employing computer vision or robotics-based approaches primarily evaluated algorithmic feasibility rather than clinical prediction performance. Cloix et al.^24^ demonstrated high environmental hazard recognition accuracy (> 90%) for stair and curb detection, while Moore et al.^26^ reported strong object-detection performance using YOLOv8 (mAP50 = 0.81).

Environmental factors consistently contributed to fall risk prediction when incorporated into AI-based models, although their representation and relative importance varied substantially by data source and modeling approach. Chen et al.^27^ demonstrated that house tidiness emerged as one of the most important environmental predictors for both falls and fall-related injuries, alongside prior fall history. Similarly, Lo et al.^21^ found that environmental hazards documented in OASIS-C assessments, such as inadequate lighting and household obstacles, contributed to improved discrimination compared with a standard clinical screening tool. Lathouwers et al.^23^ further demonstrated that environmental vulnerability indicators contributed alongside biological, behavioral, and socioeconomic factors, with each environmental predictor accounting for a comparable proportion of overall fall risk.

## Discussion

This systematic review provides a focused synthesis of AI-based fall risk prediction models that incorporate environmental factors among community-dwelling older adults, addressing a critical gap in the existing fall prevention literature. One observation from this study emerged during the search and screening stage. Despite identifying over 20,000 records through a comprehensive search, most studies were excluded because environmental factors were not incorporated into AI-based fall prediction models. Among the included studies, while personal, clinical, and functional factors appropriately formed the foundation of model development, environmental factors were typically included as part of broader predictor sets rather than being explicitly examined as distinct contributors to fall risk. These findings highlight a critical gap in AI-based fall prediction research and reveal environmental context as an underdeveloped yet potentially high-impact component.

Notably, synthesis across diverse AI applications demonstrated that when environmental features were incorporated, they consistently contributed meaningful information to fall risk prediction,^21,23,27^ either by improving model discrimination or by identifying actionable home hazards. These findings suggested that environmental factors are not peripheral but provide complementary and actionable information that enhances fall risk prediction. An important implication of these findings is that environmental factors differ from many personal or clinical predictors in that they are inherently modifiable.^30–32^ While variables such as age, chronic conditions, or prior falls primarily support risk stratification,^33,34^ environmental hazards can directly inform targeted prevention strategies. Lack of attention to the environmental context in current AI-based models may constrain both predictive insight and the translation of model outputs into actionable prevention strategies. Therefore, further study is warranted to explicitly incorporate environmental risk factors, which could strengthen the link between fall prediction and practical interventions in community settings.

Fall hazards can be identified through traditional home environmental assessments conducted by healthcare providers during home visits, which are considered as a gold-standard approach.^35–37^ However, their implementation is often constrained by substantial practical limitations. For example, comprehensive validated tools such as the *Westmead Home Safety Assessment* consist ofa 72-item checklist,^38^ require considerable time and clinical effort and may yield variable results depending on provider expertise and perspective.^39^ Additionally, access is limited for older adults in remote areas due to shortages of trained personnel, travel barriers, and economic constraints,^31,40^ In parallel, this review identified substantial heterogeneity in how environmental risk factors are represented in AI-based models, ranging from simple binary indicators (e.g., presence of handrails) to high-dimensional sensor- or vision-derived data. Such inconsistency limits model comparability, replication, and cumulative knowledge building, ultimately constraining translation into practice.^41^ Therefore, the need for AI-based approaches is highlighted as capable of supporting standardized, scalable, and objective environmental risk assessment. In this context, AI-driven methods, such as computer vision, image-based analysis, and multimodal data integration, offer promising opportunities to reduce assessment burden while enabling more standardized, precise, and usable representations of environmental risk information within fall prevention workflows. However, for real-word adoption in routine clinical practice, existing vision- and robotics have largely focused on algorithmic feasibility, have not yet demonstrated clinical performance, and have provided limited participant demographic information. Consequently, further validation across diverse clinical scenarios is necessary before clinical implementation.

From an aging and community health perspective, the limited integration of environmental context in AI-based fall prediction models has important implications for equity, real-world relevance, and healthy aging. As people age, their immediate living environments play an increasingly central role in daily functioning,^42,43^ making housing conditions and neighborhood infrastructure key determinants of fall risk.^44–46^ These environmental conditions vary widely across populations and are closely linked to lifelong socioeconomic circumstances, geographic location, and access to supportive resources.^44–46^ Consequently, AI models that insufficiently account for environmental risk may systematically overlook or underestimate key sources of vulnerability among community-dwelling older adults, particularly those aging in place within resource-limited settings. Such omissions risk reinforcing cumulative disadvantages in later life by limiting accurate risk identification and delaying preventive action, even when overall model performance appears acceptable. Incorporating environmental context into AI-based fall prediction models can therefore move these tools beyond risk stratification toward more context-aware and actionable insights, enabling earlier identification of modifiable hazards and supporting targeted interventions that promote safer aging in place and more equitable fall prevention across diverse aging populations.

This systematic review has several limitations. First, although a comprehensive search strategy was employed across multiple databases, relevant studies may have been missed due to publication bias, indexing limitations, or exclusion criteria such as restriction to English-language publications and peer-reviewed articles, potentially excluding gray literature. Second, the small number of eligible studies reflects the emerging nature of AI-based fall prediction models that explicitly incorporate environmental factors, limiting the ability to draw definitive conclusions or conduct quantitative synthesis. Third, fall-related outcomes varied across studies, including any falls, recurrent falls, and fall-related injuries, with varying follow-up durations. This variability complicates comparison across models and may influence reported performance metrics. Fourth, several studies did not report external validation or relied on single datasets, raising concerns about model generalizability. The extent to which models incorporating environmental factors perform consistently across different populations and settings remains uncertain. Fifth, most included studies were conducted in high-income countries, potentially limiting the applicability of findings to low- and middle-income settings or to culturally diverse housing environments where environmental risks and living conditions may differ substantially. Finally, while some studies identified environmental hazards or demonstrated improved predictive performance, few evaluated whether incorporating environmental factors altered fall prevention interventions or reduced fall incidence, limiting conclusions about real-world effectiveness.

Despite several limitations, this study has a number of important strengths. First, this study is the first systematic review to specifically examine how environmental factors are incorporated into AI-based fall risk prediction models. In doing so, we address a critical gap in the existing literature, which has largely focused on individual- and functional-level predictors. By isolating the environmental dimension, this review advances understanding of how modifiable home-related risks are currently conceptualized and operationalized within AI-based approaches. Second, this study provides a broad and integrative perspective on environmental risk representation by synthesizing evidence across diverse AI paradigms ranging from traditional machine learning models using structured data to computer vision and robotics-based approaches. Finally, this review provides clear evidence that environmental factors contribute meaningful information to AI-based fall risk prediction models.

## Future Implication

Future research on AI-based fall prediction should address several important gaps identified in this review.

(1) There is a clear need for standardized definitions and measurement frameworks for environmental risk factors, as current studies vary widely in how environmental hazards are conceptualized, assessed, and represented in models. Without greater consistency, comparison across studies and translation into practice remain limited.

(2) Multimodal AI models that integrate clinical, functional, and environmental data are needed to more fully capture the multifactorial nature of fall risk. Falls often occur as the result of dynamic interactions among individual characteristics, functional limitations, and environmental hazards, rather than from any single factor alone. While many existing models emphasize individual- and functional-level predictors, incorporating environmental information alongside these factors may improve predictive performance and clinical relevance. Given that many environmental hazards are inherently visual and context-dependent, image-based or computer vision–driven approaches may be particularly valuable for identifying home environmental risks that are difficult to capture through structured questionnaires or clinical assessments alone. Advances in image recognition, object detection, and depth sensing offer promising opportunities to enhance environmental risk detection in real-world home settings, ultimately informing targeted interventions to reduce fall risk.

(3) Environmental characteristics may vary significantly across different geographic regions and cultural contexts. Future research could further explore adaptive modeling approaches for environmental factors in multicultural settings, aiming to develop models that generalize better across regions. In addition, investigating potential fall risks arising from the interaction between cultural behaviors and environmental contexts may contribute to more inclusive, culturally sensitive, and accurate fall risk prediction.

(4) Environmental features were generally assessed at a single time point and treated as static predictors. This approach does not reflect the dynamic nature of home environments, where hazards may change over time due to health decline, behavior adaptation, seasonal conditions, or home modifications. Future AI models may benefit from longitudinal or continuously updated environmental data to better reflect real-world fall risk trajectories.

(5) Future AI-based fall prediction tools should prioritize scalability, usability, and real-world applicability, ensuring that models can be feasibly deployed in community settings and adapted to diverse home environments. Approaches that leverage mobile devices, smart home technologies, or automated image-based assessments may help bridge the gap between predictive modeling and actionable fall prevention strategies.

## Conclusions

This review shows that environmental factors remain infrequently emphasized in AI-based fall prediction models, despite their established relevance to fall risk among community-dwelling older adults. Environmental context provides complementary and actionable information that supports the need for more standardized, context-aware AI approaches to equitable and effective fall prevention.

## Figures and Tables

**Table 1:** Overview of Included Studies

Author (Year of publication)	Study region	Study design	Aim	Date Source	Participants characteristics
Chen et al. (2023)^27^	China	Cohort study	To build prediction models for falls and fall-related injuries among community-dwelling older adults in China	China Health and Retirement Longitudinal Study (CHARLS), a national cohort of middle-aged and older Chinese adults (mainly ≥ 45 years) across 28 provinces. Launched in 2011 with follow-up in 2013, 2015, and 2018. This study used data from the latest two waves (2015 and 2018).	Recruitment: Not applicable. Inclusion criteria: Not applicable. Exclusion criteria: (1) aged < 60 years, (2) living in a care home, (3) did not participate in 2015 and 2018 follow-ups, (4) incomplete information about falls and blood indices, and (5) > 10% missing data in individual variables. Total participants: 5,818.
Cloix et al. (2016)^24^	Switzerland	Other: Technology Evaluation Study	To evaluate a low-complexity algorithm to detect descending stairs and curbs of any shape, specifically designed for low-power real-time embedded platforms	The cameras were located at 78 cm height with a tilted angle of 35°. Images were captured at 512 × 384 and 640 × 480 pixel resolution with the stereo camera and the Red Green Blue-Depth (RGBD) sensor.	The assessment of our approach was carried out using thirteen scenes of descending stairs and curbs. Recruitment: Not applicable. Inclusion criteria: Not applicable. Exclusion criteria: Not applicable. Total participants: Not applicable.
Du et al. (2014)^22^	USA	Other: Engineering Feasibility and Prototype Validation Study	To develop a robot-based system for in-home environment screening that supports both manual and autonomous fall risk assessment.	Real-time sensor data collected by a mobile robot (Turtlebot2) in a simulated in-home environment.	Recruitment: Not applicable. Inclusion criteria: Not applicable. Exclusion criteria: Not applicable. Total participants: Not applicable.
Lathouwer et al. (2022)^23^	Belgium	Cohort study	To identify fall risk factors using a quality-of-life questionnaire covering biological, behavioral, environmental, and socioeconomic domains.	Questionnaire developed by the Belgian Ageing Studies research group of the Vrije Universiteit Brussel.	Recruitment: Stratified random sampling (by sex and age) from census data of community-dwelling adults aged ≥ 60 in Belgian municipalities. Inclusion criteria: Community-dwelling older adults aged ≥ 60 living in Belgium. Exclusion criteria: Not applicable. Total participants: 33,346 community-dwelling older adults; 84 questionnaire items excluded due to missing data, resulting in 24 input features and 33,346 valid entries.
Lo et al. (2019)^21^	USA	Cross sectional study	To devise a machine learning pipeline using existing home health care data to predict fall risk among older adults, with the goal of building clinical decision support tools for fall prevention	Outcome and Assessment Information Set (OASIS-C) – mandatory assessment for home health care patients; Electronic Health Records (EHR) – supplemental demographic data.	Recruitment: Not applicable (retrospective EHR-based study). Inclusion criteria: Age ≥ 65 years; available OASIS-C assessment data and MAHC-10 fall risk assessment data. Exclusion criteria: Not applicable. Total participants: 59,028 unique patients.
Millet et al. (2023)^29^	Spain	Cohort study	To predict recurrent falls in the older population using machine learning techniques, with the aim of reducing the number of falls and their consequences.	Getafe University Hospital’s Geriatric Falls Unit (data obtained from the Hospital’s Electronic Health Records) between January 2017 and December 2021. Older adults who experienced a fall requiring medical attention were referred to this unit for personalized treatment and monitoring to prevent repeated falls.	Recruitment: Not applicable. Inclusion criteria: Adults who were treated at the Geriatric Falls Unit for at least one fall (mean age 80.3±7.7). Exclusion criteria: Not applicable. Total participants: 304 older adults.
Moore et al. (2024)^26^	UK	Other: A mixed-method study	To develop an AI algorithm using wearable video-based eye-tracking and IMU gait data, and to explore how people with Parkinson’s disease perceive the use of video and AI technologies in daily life for understanding their fall risk.	For developing the AI model, pre-trained MS COCO weights were initialized and fine-tuned on a local dataset (80:20 split). Ten hours of local video data (> 1500 frames) were manually extracted and annotated using the LabelImg tool, with 4 categories and 18 classes selected for relevance to fall risk or privacy. A total of 1542 frames were selected and annotated, including: (1) scripted route data from 7 young adults covering 10 interior and 10 exterior environments; (2) 240 manually extracted frames from 3 PwPD, ensuring unseen data for testing; and (3) 4 additional first-person-view exterior videos (~ 240 min) downloaded under CC-BY licence and annotated.	Recruitment: PwPD participants were recruited locally through networks within Northumbria University. Seven young adults (6M:1F, 23–32 years) were recruited to wear video glasses solely for generating AI training data; young adults were recruited through word of mouth. Inclusion criteria: For PwPD: Clinical diagnosis of Parkinson’s disease; Prior experience in wearable-gait research; Familiarity with technology (e.g., smartphones, tablets, applications); Willingness to attend focus groups and consent to audio-recording; English-speaking and literate. For young adults: Not formally specified beyond age and functional independence. Exclusion criteria: For PwPD: Significant cognitive impairment. For young adults: Any functional impairments. Total participants: Video dataset included frames from 7 young adults and 3 PwPD; focus group size was n = 4.
Panyakaew et al. (2021)^28^	Thailand	Cross sectional study	To explore the prediction of falling in Parkinson’s disease patients using a machine learning-based approach.	Recruited from the Chulalongkorn Center of Excellence for Parkinson’s Disease and Related Disorders, Faculty of Medicine, Chulalongkorn University, Thailand, between January and December 2019.	Recruitment: Patients with a clinical diagnosis of PD with Hoehn & Yahr (H&Y) stage 1–4, with or without a history of falls, were recruited from the same center (www.chulapd.org) between January and December 2019. Inclusion criteria: (1) Clinical diagnosis of PD based on standard diagnostic criteria; (2) Hoehn & Yahr (H&Y) stage 1–4; (3) able to ambulate within community residences; (4) able to follow simple commands with a score ≥ 21 on the Thai version of the Mini-Mental Status Examination (MMSE); and (5) able to complete the ABC-16 scale independently. Exclusion criteria: (1) Hoehn & Yahr stage 5; (2) coexisting disorders contributing to postural instability and falls, including stroke, ataxia, neuropathy, and visual, vestibular, or proprioceptive problems; (3) patients taking sedative medications. Total participants: 305.
Pérez-Ros et al. (2019)^25^	Spain	Other: Cohort trial nested case-control design	To determine the predictive factors for falls in functionally independent community dwelling older adults.	Data was collected from a prospective, longitudinal study of 374 community-dwelling older adults aged ≥ 70 years in La Ribera, Valencia, Spain, recruited from primary care centers between December 2013 and May 2014, with a 12-month follow-up to assess falls.	Recruitment: A publicity strategy was used from December 2013 to May 2014, including posters and notifications in retirement centers, meetings with managing authorities of retirement associations and centers, and telephone calls to individuals enrolled in primary care centers. Family telephone calls were also used to reduce dropouts among individuals lacking the physical means to attend fall-prevention education sessions. Inclusion criteria: (1) Aged ≥ 70 years; (2) Barthel Index ≥ 60 (functionally independent); (3) Independent walking (with or without technical aids, but not assisted by another person); (4) Resident in the La Ribera region. Exclusion criteria: (1) Life expectancy < 6 months; (2) Blindness or deafness; (3) Serious psychiatric illness (schizophrenia, major depression, bipolar disorder, panic disorder); (4) Moderate to severe cognitive impairment (MEC-Lobo < 24); (5) Did not sign informed consent. Total participants: 374.

**Table 2:** AI-Based Fall Risk Models and Environmental Risk Factors

Author (Year of publication)	Type of AI algorithm	Environmental Risk Factors	Non-Environmental Risk Factors	Model Performance	Limitation
Chen et al. (2023)^27^	Machine learning AI algorithm: logistic regression (LR); support vector machine (SVM); random forest (RF); adaptive boosting (AdaBoost); light gradient boosting machine (LightGBM)	Structure of building; handicapped facilities; kitchen availability; flush toilets; cooking fuel type; Internet availability; house tidiness; house temperature Measures of environmental risks: inquiry-based survey measures	Biological, behavioral, and socioeconomic predictors including sex, vision, diabetes, liver disease, memory-related disease, disabilities (brain, vision, physical), ADL, IADL, experience of falling, hip fracture history, smoking, alcohol use, sleep duration, medications (antihypertensive, lung, heart, kidney, memory, arthritis), depressive symptoms, dental care, relationship with children, health satisfaction, systolic BP, walking speed, hand strength, white blood cell count, blood urea nitrogen, glucose; injury model includes marital status, stroke, dyslipidemia medication, digestive medication, income, life satisfaction, lung function, abdominal obesity	The LR model showed good performance with an AUC-ROC of 0.739 (sensitivity 0.707) for falls and an AUC-ROC of 0.757 (sensitivity 0.654) for fall-related injuries. Fall risk impact: Baseline falling experience was the most important predictor. A history of falls was the strongest predictor of future falls, and house tidiness was an important environmental predictor in both fall and fall-related injury models.	Variables were treated as categorical; causal inference was not possible; predictors were only measured at baseline, without accounting for changes over time.
Cloix et al. (2016)^24^	Computer vision for stairs and curbs detection AI algorithm: Binary prediction algorithm (Algorithm 1) using upper/lower depth map (Dp) from stereo images; Three-class prediction algorithm (Algorithm 2) using full depth map (D) divided into sub-depth maps; thresholds TG/TR and TGi/TRi applied to ground distance and ratio of valid pixels Environmental risks identified:	Stairs and curbs Measures of environmental risks: depth difference from stereo disparity; ratio of pixels below ground level; spatial location of drop (upper vs. lower half of image); predicted “danger zone” proximity using trapezoid projection	Not applicable	The system distinguished more than 94% dangerous scenes from safe scenes with an overall recognition rate of 91% at very low resolution, operating in real time and robust to indoor/outdoor lighting conditions. Fall risk impact: Not applicable.	HDR cameras can better capture scenes under bright sunlight to avoid saturation that causes stereo image matching to fail, and DSPs may assist with motion blur removal before disparity computation to improve the SAD stereo image matching.
Du et al. (2014)^22^	Computer vision, robotics, and shared control systems AI algorithm: Gmapping (Rao-Blackwellized Particle Filter SLAM); OctoMap (3D mapping using octrees); costmap-based obstacle avoidance planning; AMCL (Adaptive Monte Carlo Localization)	Poor lighting; cluttered or narrow spaces; obstructed walkways; limited visibility (telepresence) Measures of environmental risks: luminosity (lux) thresholds: ≥401 = bright, 201–400 = medium, < 200 = dark; lighting condition displayed on web interface (green = bright, yellow = medium, red = dark)	Not applicable	Model performance: Not reported. Fall risk impact: Not applicable.	The system is a prototype and requires major improvements, including automation of assessment tasks, addition of object recognition functions for independent hazard detection, and better error handling and recovery methods to ensure reliability and stability for use in real world.
Lathouwer et al. (2022)^23^	Machine learning AI algorithm: Random Forest model	Housing issues, housing change, environmental vulnerability Measures of environmental risks: not reported	24 variables including biological (loneliness, sex), behavioral (physical vulnerability, physical effort, physical activity, mental activity, help required, help available, mode of transportation, psychological vulnerability), and socioeconomic factors (age class, education, civil status, surrounding density, homeownership, home type, number of children and grandchildren, insecurity, neighborhood organization, social vulnerability)	The model reached an average of 73% accuracy. Fall risk impact: The classification model identified 24 predictors of falling (2 biological, 8 behavioral, 11 socioeconomic, and 3 environmental factors), each contributing 4.5–6.5% to the overall fall risk.	Further work is needed to improve data quality, address multicollinearity, explore additional ML methods (e.g., SVM, neural networks), and enhance accuracy through hyperparameter tuning and larger datasets.
Lo et al. (2019)^21^	Machine learning AI algorithm: Random Forest	Clutter or obstacles; inadequate lighting; unsafe flooring; lack of assistive devices or improper use Measures of environmental risks: assessed via OASIS-C structured items recorded by home health care clinicians during mandatory assessments	age; sex; language group; borough of residence; number and severity of diagnoses; pain levels; cognitive impairment; visual impairment; frequency of assistance with ADL/IADL; therapy visit frequencies; living situation	The OASIS model achieved a balanced accuracy of 0.62, an AUC-ROC of 0.67 (95% CI: 0.66–0.68), and an average precision of 0.10, outperforming the baseline MAHC-10 scoring system. Fall risk impact: A random forest model using OASIS-C and EHR data provided improved fall risk prediction compared with the MAHC-10 tool, with age, severity of diagnoses, therapy visits, pain, and ADL/IADL assistance identified as top predictors supporting individualized fall-prevention strategies.	The study relied on data from a single home care agency, and model precision remained low due to highly imbalanced outcomes; nearly 95% of patients in the cohort had no reported falls.
Millet et al. (2023)^29^	Machine learning + Natural language processing AI algorithms: Random Forest; Decision Tree; Logistic Regression; LightGBM; Support Vector Machine (SVM); K-Nearest Neighbors (KNN)	Stairs at home; teleassistance; home accessibility; elevator; stairs management (from selected 25 features) Measures of environmental risks: Not clarified	BMI; weight; age; gait speed; height; standing balance; walking cane; grip strength; sitting balance; foot support; teleassistance; lift; antiplatelet drugs; cognitive impairment; anticoagulants; hip fracture; dizziness; depressive syndrome; fear; memory loss; stable turns; disorientation	Best performance achieved by the Bagging ensemble using Random Forest, which attained 75.8% accuracy, 70.0% sensitivity, 80.5% specificity, and an AUC of 75.3%. Among base models, Random Forest alone performed strongly with 76.9% accuracy and 85.0% specificity, though with lower sensitivity (65.5%). Ensemble methods generally improved robustness and balance across metrics. Fall risk impact: Accurate prediction of recurrent falls in older adults using routine clinical data.	Reliance on retrospective data from a single hospital, the semi-structured nature of clinical notes requiring extensive NLP preprocessing, relatively low model sensitivity, and the absence of dynamic risk updates or validation in prospective or interventional settings.
Moore et al. (2024)^26^	Deep learning model AI algorithm: YOLOv8 object detection algorithm	Stairs; doorway; shower; sink; toilet; table; bed; signage; chair; animal; wet surface; mat/rug/carpet; obstacle (generic catch-all for potential obstructions); raised curbs Measures of environmental risks: Not applicable	Vehicle	Using the collected dataset, the YOLOv8 algorithm was trained for a course of 100 epochs converging at epoch 69 within a timeframe of 4 h. The models achieved a best validation mAP50 of 0.81 at epoch 69, showcasing the potential of this algorithm within real-world deployment. Fall risk impact: NA	Only a small number of PwPD were recruited, and a limited dataset was curated as part of the pilot study. The number of participants for the focus group was small too and they were recruited based on their prior experience of participating in wearable gait research and their familiarity with technology. The focus group participants may have been biased toward the acceptance of technology as they were recruited by purposive sampling to have a good understanding and/or appreciation of commercial technology.
Panyakaew et al. (2021)^28^	Type of AI: Machine learning AI algorithm: XGBoost models for predicting (1) falls (fallers vs. non-fallers) and (2) recurrent falls (recurrent vs. non-recurrent fallers); SHAP used for model interpretability	High-risk daily activities including sweeping the floor (ABC-16 item 7); reaching on tiptoes (item 5); walking in a crowded mall (item 12); walking across a parking lot (item 10); getting in/out of a car (item 9); walking up/down stairs (item 2); walking up/down a ramp (item 11) Measures of environmental risks: Activities-Specific Balance Confidence Scale (ABC-16)—self-reported confidence(0–100%) in 16 daily activities	H&Y stage 3, disease duration, age, PD subtype, Postural Instability and Gait Disorder (PIGD), wearing-off, dyskinesia, use of dopamine agonists, use of COMT inhibitors	Falls model accuracy: 72% (p = 0.001) Recurrent falls model accuracy: 81% (p = 0.02) Fall risk impact: The study successfully predicted fallers (72%) and recurrent fallers (81%). Top predictive environmental activities included: Sweeping the floor, Reaching on tiptoes, Walking in a crowded mall, Walking across a parking lot, and Getting in/out of a car.	Exclusion of neurological comorbidities; lack of details on anti-parkinsonian medication use; PD patients had relatively normal cognition with no neuropsychiatric/non-motor details. Only total MMSE score was available, with no breakdown of cognitive domains or executive function. Retrospective fall reporting; exclusion of patients with MMSE < 21; no objective balance measures; medication details lacking.
Pérez-Ros et al. (2019)^25^	Machine learning AI algorithm: Binary logistic regression models	Lack of stair handrails; poor stair design; lack of bathroom grab bars; dim lighting or glare; obstacles and tripping hazards; slippery or uneven surfaces; improper use of assistive devices Measures of environmental risks: recorded as presence/absence of specific hazards via nurse-administered geriatric assessment at baseline	Advanced age (≥ 80 years); previous falls; muscle weakness; gait and balance problems; poor vision; postural hypotension; chronic conditions (osteoarthritis, diabetes, stroke, Parkinson’s disease, incontinence, dementia); fear of falling (mFES); previous fractures; obesity (BMI ≥ 30 kg/m^2^); hearing problems; anxiety-depressive syndrome; medication use (alpha-blockers, benzodiazepines, beta-blockers, CNS-acting drugs); polypharmacy	Isolated falls model: Sensitivity 10%, Specificity 98.2%, PPV 75.2%, NPV 77.5%, Nagelkerke R^2^ = 47%, Overall correct classification = 77%. Recurrent falls model: Sensitivity 15.2%, Specificity 98.8%, PPV 58.8%, NPV 92.3%, Nagelkerke R^2^ = 69.4%, Overall correct classification = 91.4%. Fall risk impact: Predictive models identified that prior falls and use of alpha-blockers predicted isolated falls, while previous fractures, obesity (BMI ≥ 30 kg/m^2^), and use of benzodiazepines and beta-blockers predicted recurrent falls in independent older adults. These findings highlighted modifiable risk factors that may inform fall-prevention strategies.	Drug-related data reflected only commonly prescribed pharmacotherapeutic groups; drug doses were not documented. Time, weather, and location of falls were not recorded. Follow-up was limited to 12 months. Self-reported monthly falls may be biased due to missing information. Medication adherence was not assessed.
